# Transgenic *Eimeria mitis* expressing chicken interleukin 2 stimulated higher cellular immune response in chickens compared with the wild-type parasites

**DOI:** 10.3389/fmicb.2015.00533

**Published:** 2015-06-02

**Authors:** Zhuoran Li, Xinming Tang, Jingxia Suo, Mei Qin, Guangwen Yin, Xianyong Liu, Xun Suo

**Affiliations:** ^1^State Key Laboratory of Agrobiotechnology, China Agricultural University, BeijingChina; ^2^National Animal Protozoa Laboratory, College of Veterinary Medicine, China Agricultural University, BeijingChina; ^3^The High School attached to Tsinghua University, BeijingChina; ^4^Key Laboratory of Animal Epidemiology and Zoonosis of Ministry of Agriculture, China Agricultural University, BeijingChina

**Keywords:** transgenic *Eimeria mitis*, chicken interleukin 2, reproductive potential, cellular immune response, ELISPOT

## Abstract

Chicken coccidiosis, caused by *Eimeria* sp., occurs in almost all poultry farms and causes huge economic losses in the poultry industry. Although this disease could be controlled by vaccination, the reduced feed conservation ratio limits the widespread application of anticoccidial vaccines in broilers because some intermediate and/or low immunogenic *Eimeria* sp. only elicit partial protection. It is of importance to enhance the immunogenicity of these *Eimeria* sp. by adjuvants for more effective prevention of coccidiosis. Cytokines have remarkable effects on the immunogenicity of antigens. Interleukin 2 (IL-2), for example, significantly stimulates the activation of CD8+ T cells and other immune cells. In this study, we constructed a transgenic *Eimeria mitis* line (EmiChIL-2) expressing chicken IL-2 (ChIL-2) to investigate the adjuvant effect of ChIL-2 to enhance the immunogenicity of *E. mitis* against its infection. Stable transfected EmiChIL-2 population was obtained by pyrimethamine selection and verified by PCR, genome walking, western blotting and indirect immunofluorescence assay. Cellular immune response, *E. mitis*-specific IFN-γ secretion lymphocytes in the peripheral blood mononuclear cells, stimulated by EmiChIL-2 was analyzed by enzyme-linked immunospot assay (ELISPOT). The results showed that EmiChIL-2 stimulated a higher cellular immune response compared with that of the wild-type parasite infection in chickens. Moreover, after the immunization with EmiChIL-2, elevated cellular immune response as well as reduced oocyst output were observed These results indicated that ChIL-2 expressed by *Eimeria* sp. functions as adjuvant and IL-2 expressing *Eimeria* parasites are valuable vaccine strains against coccidiosis.

## Introduction

Infections by *Eimeria* sp. occur in almost all poultry farms and cause approximately £2 billion losses in the poultry industry 1 year (Shirley et al., 2005; Suo et al., 2006). Vaccination with either the virulent (Coccivac^®^ and Immucox^®^) or the attenuated (Paracox^®^ and Livacox^®^) live parasites formulations has been considered the most efficient means for the protection of breeder and layer flocks from *Eimeria* sp. infection (Williams, 1998; Shirley et al., 2005; Suo et al., 2006). When chickens are inoculated with a live anticoccidial vaccine, the species within the vaccine will finish their life cycle in the host intestine and their offspring oocysts will be excreted into the environment (litter) together with feces. Immunity against re-infection by *Eimeria* species will be boosted when vaccinated chickens “eat” these offspring oocysts (Williams, 1998; Shirley et al., 2005). The cell-mediated immunity (CMI) plays a major role in the host protection against coccidiosis and requires reinfections to become solid after vaccination (Danforth, 1998; Chapman, 2000). For *Eimeria* species with high immunogenicity, immunity boosted by the first round oocysts will be solid enough to prevent further infection by large quantity of oocysts in the litter, but for those with low or intermediate immunogenicity, immunity boosted will not be solid enough and re-infection with large quantity of oocysts will occur, and the large quantity of newly invaded parasites will produce damage in the intestine and negatively influence absorbance of feed, resulting in bad feed conversion limiting the wide use of anticoccidial vaccines in broilers (Jeffers, 1975; Shirley et al., 2005; Chapman et al., 2013). Therefore, the enhanced immunogenicity of some *Eimeria* sp. such as *Eimeria mitis* through transfection of adjuvant molecules is hypothesized to elicit a higher cellular immune response and eliminate the intracellular pathogens rapidly, a strategy that can be utilized for the development of an ideal, novel and alternative coccidiosis vaccine.

Interleukin 2 (IL-2), produced by helper T cells, is a growth factor that plays a major role in the expansion and differentiation of CD4+ and CD8+ effector T cells both *in vivo* and *in vitro* (Pardoll, 2002; Blachere et al., 2006; Rochman et al., 2009), and in the activation of N K and LA K cells (Grimm et al., 1982; Trinchieri, 1989). In a mouse model, the exogenous IL-2 added to a peptide plus CpG-containing oligodeoxynucleotides (CpG ODN) vaccination regimen dramatically increased the peptide-vaccine-elicited CD8+ T cell responses 221-fold compared with those after CpG ODN and peptide vaccination in B16F1 melanoma infection (Addison et al., 1998). Recently, the mucosal immunization of mice with recombinant *Lactococcus lactis* NZ9000, expressing the UreB-IL-2 protein, elicited more anti-UreB antibodies that specifically bounded to the purified *Helicobacter pylori* UreB protein (Zhang et al., 2014). Thus, more research is being conducted to confirm the adjuvant effect of IL-2 in enhancing immunogenicity of live vaccine strains (Addison et al., 1998; Zhang et al., 2014).

Here, we hypothesized that chicken IL-2 (ChIL-2), applied as an adjuvant, enhanced the *Eimeria*-specific cell-mediated immune response in chickens. To verify our hypothesis, we choose to conduct the experiments with *E. mitis*, an intermediately immunogenic *Eimeria* species, to locally express ChIL-2. Our results showed that the transgenic *E. mitis* expressing ChIL-2 (EmiChIL-2) elicited a higher cellular immune response than the wild-type *E. mitis* infection in chickens. Thus, it is encouraging that other transgenic other *Eimeria* sp., which also express ChIL-2, could be successfully implemented as an alternative coccidiosis vaccine for wide use in the poultry industry.

## Materials and Methods

### Ethics Statement

Our research with animals was approved by the Beijing Administration Committee of Laboratory Animals and performed in accordance with the China Agricultural University Institutional Animal Care and Use Committee guidelines.

### Parasite and Animals

*Eimeria mitis* (Zz strain), used in this study was maintained by passaging in coccidian-free, 2–5-weeks-old AA broilers. The procedures for collection, purification, and sporulation were carried out as previously described (Long et al., 1976).

Three-weeks-old SPF chickens were purchased from Merial Animal Health Co., Ltd. (Beijing, China) and were fed a pathogen-free diet and water *ad libitum*.

### Plasmid Construction

Total RNA was isolated from the spleen lymphocytes of one 3-weeks-old SPF chicken by using the TRIzol reagent (Invitrogen, USA). cDNA was synthesized through the utilization of random primers and a High Capacity cDNA Reverse Transcription Kit (Applied Biosystems, USA). According to the ChIL-2 sequence of *Gallus gallus* (GeneBank Accession number: AF000631.1), the open reading frame of ChIL-2 was amplified by PCR via the use of ChIL-2-F/ChIL-2-R (**Table 1**). The *Toxoplasma gondii* dihydrofolate reductase-thymidylate synthase (DHFR-TSm2m3) gene and the enhanced yellow fluorescent gene (EYFP) were amplified by PCR through the use of DHFR-F/DHFR-R and EYFP-F/EYFP-R from pEtADA and pMIC-EYFP/ACTss-RFP (Huang et al., 2011; Yin et al., 2011), respectively. A fused DHFR-EYFP gene was obtained by overlapping PCR. All PCR amplifications were performed by using the high fidelity thermostable *Pfu* DNA polymerase to reduce the mutation frequency.

**Table 1:** Primers used in this study.

Primer name	Primer sequences (5′–3′)^a^	Restriction enzyme name
DHFR-F	GGTACCATGCAGAAGCCGGTGTGTCTGGTC	Kpn I
DHFR-R	CAGCTCCTCGCCCTTGCTCACCATGGTGGCGAC	**–**
	AGGATCCAAGACAGC	
EYFP-F	GCTGTCTTGGATCCTGTCGCCACCATGGTGAGC AAGGGCGAGGAGCTG	**–**
EYFP-R	CCTAGGTCAAAGCTTCTTGTACAGCTCGTC	Avr II
ChlL-2-F	ATGATGTGCAAAGTACTGATCT	
ChlL-2-1	GTAGCTCGCGCCATGAACGGTCCTTTGATGATGT GCAAAGTACTGATCT	**–**
ChlL-2-2	GTGTTCGTGGTCTTCGCTGTCTTTGGTGTAGCTC GCGCCATGAACGGTCCTTTG	**–**
ChlL-2-3	ACCGGTATGGCTTTACCATTGCGTGTTTCGGCCA	Age I
	CGGTGTTCGTGGTCTTCGC	
ChlL-2-R	CCGCGGTTATTTTTGCAGATATCTCAC	Sac II
SP-1	GCTTGCAGCACTTCAGACACTCAA	**—**
SP-2	AAAGACAGAAGTGCCAGCAGCAG	**—**
SP-3	CTGCAACATTCAGTGACTTAGCCG	**—**

*^a^Restriction enzyme sites in the sequence are underlined.*

**FIGURE 1:**
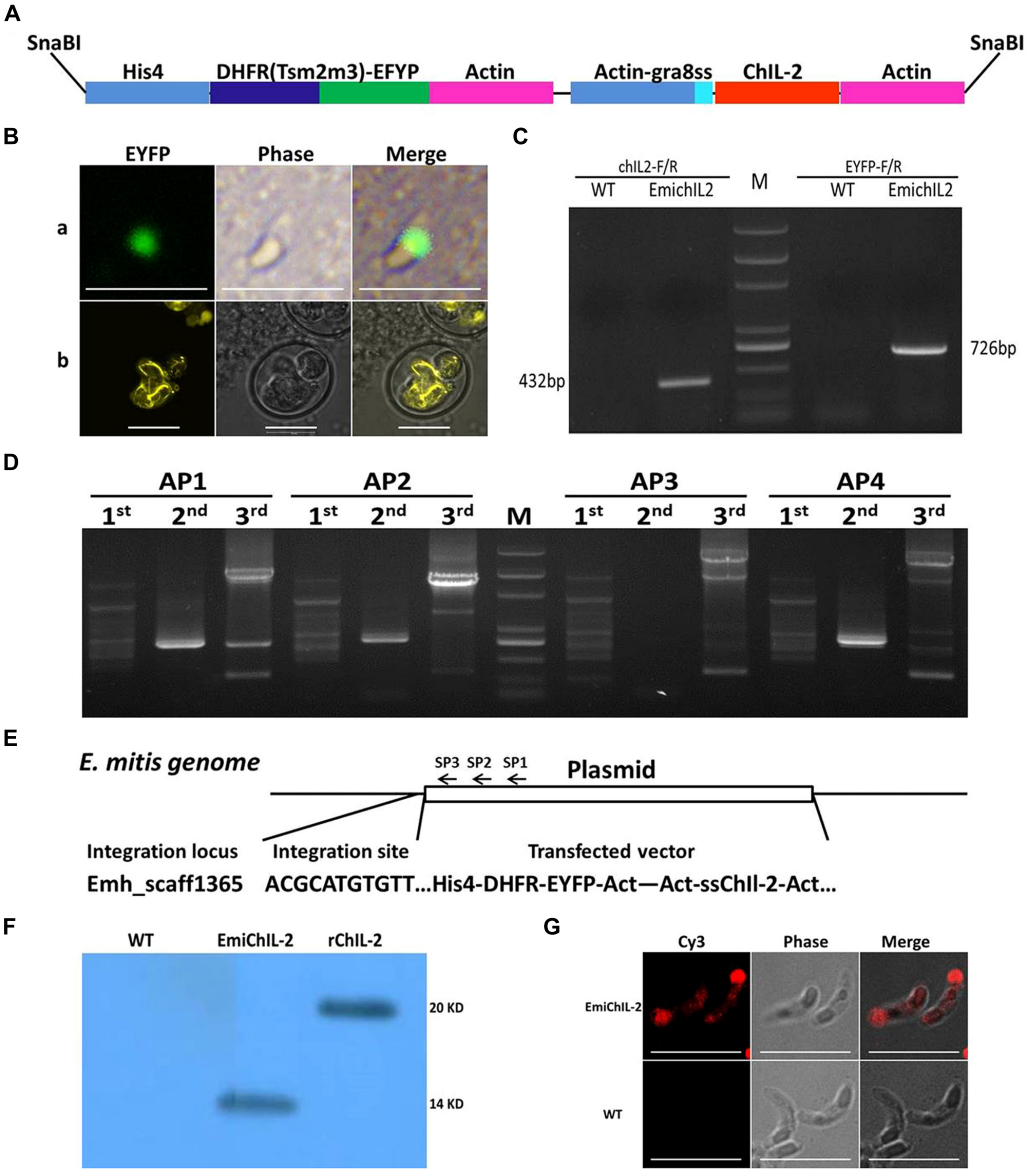
**Construction of transgenic *Eimeria mitis* expressing secreted chicken IL-2 (ChIL-2). (A)** Schematic of double-cassette expression vectors. The selection gene [DHFR-Ts-enhanced yellow fluorescent gene (EYFP)] and ChIL-2 were driven by the histone 4 and actin promoter, respectively. Signal sequences (ss) from *T. gondii* GRA 8 regulated the secretion of ChIL-2. **(B)** Both the transiently transfected *E. mitis* sporozoites (a) and the stable transfected EmiChIL-2 (b) were expressing EYFP. **(C)** Genomic DNA from EmiChIL-2 was amplified with the primers ChIL-2-F and ChIL-2-R (giving a 432 bp product) to verify the recombination of ChIL-2, and the primers EYFP-F and EYFP-R (giving a 726 bp product) to confirm the recombination of EYFP as a positive control, genomic DNA from wild type *E. mitis* was used as a control. **(D)** Genomic DNA from EmiChIL-2 was amplified with arbitrary degenerate primers (AP 1, AP 2, AP 3, and AP 4) and specific primers [SP 1, SP 2, and SP 3 (**Table 2**)] from histone 4 promoter by thermal asymmetric interlaced PCR, and the products from the third-round PCR were cloned into pEasy-T1 vector for sequencing. **(E)** One integration site (Emh_scaff1365) was confirmed by BLAST from more than 50 clones in the *E. mitis* GeneDB. **(F)** Oocysts antigens extracted from EmiChIL-2 reacted with the poly antibody against ChIL-2 producing a clear band with a size of approximately 14 kd to verify ChIL-2 expression by WB. Recombinant ChIL-2 (with two His 6 tag) and the wild-type *E. mitis* oocysts antigens were used as a positive and negative control, respectively. **(G)** EmiChIL-2 sporozoites reacted with the poly antibody against ChIL-2 to confirm the localization of ChIL-2 by IFA, and the wild-type *E. mitis* sporozoites were utilized as a control. Bar = 10 μm.

The double expression-cassette plasmid, pHDEAAssChIL-2A (**Figure 1A**), was constructed based on pHIS-EYFP/ACT-RFP (Yin et al., 2011). Briefly, the EYFP gene of pHIS-EYFP/ACT-RFP was replaced by DHFR-EYFP gene following the *RFP* gene was replaced by ssChIL- 2 gene amplified by three round PCR by the use of the primers ChIL-2-R and ChIL-2-1, ChIL-2-2 and ChIL-2-3, respectively. A signal sequence (ss) was obtained from *T. gondii* GRA 8, which functionally regulates ChIL-2 secretion (Shi et al., 2009; Zou et al., 2009; Yin et al., 2011). The plasmid DNA was linearized by the SnaB I restriction enzyme, which released the two expression cassettes from the backbone of the plasmid (**Figure 1A**).

### Transfection and Selection of EmiChIL-2

For nucleofection of sporozoites, 10 million *E. mitis* sporozoites, which had been freshly purified through a DE-52 cellulose column and 10 μg linearized DNA plasmid together with 5 μl SnaB I were subjected to Nucleofector (Clark et al., 2008; Yin et al., 2011). After nucleofection (Program U-033, AMAXA, Switzerland), the sporozoites were divided into two equal parts, one of which was inoculated into cultures of primary chicken kidney cells (PCKCs) in 25 cm^2^ flasks (Corning, Costar, USA). The transient transfection ratio *in vitro* was observed by a fluorescence microscope (Olympus IX71, Tokyo, Japan). The other part of the transfected sporozoites were inoculated equally into the ileocecal opening of five 2-days-old chickens via the cloaca for stable transfection selection. Eighteen hours after the inoculation, the chickens began a standard diet, supplemented with 150 ppm pyrimethamine (Sigma, USA; Clark et al., 2008). Oocysts from feces excreted 5–10 days post infection (dpi) were collected, and sporulation was performed for next generation selection, as described before (**Table 2**; Long et al., 1976).

**Table 2:** Stable transfected EmiChIL-2 selection.

Reporter	Generation	Inoculate dosage^a^ (Oocysts/bird)	% Parasite expressing	Oocysts output/bird	Next selection^b^
EYFP	1	1 × 10^6^	1.3	1 × 10^3^	Drug
	2	5 × 10^3^	15.7	9.3 × 10^6^	Drug
	3	5 × 10^3^	35.8	1.2 × 10^7^	Drug
	4	5 × 10^3^	50.5	1.4 × 10^7^	Drug
	5	5 × 10^3^	80.3	1.1 × 10^7^	Drug
	6	5 × 10^3^	92.3	1.5 × 10^7^	–
	7	5 × 10^3^	91.7	2.7 × 10^7^	–

*^a^The first passage 2-days-old AA broilers were inoculated with transfected *Eimeria mitis* sporozoites via the cloacal route. The next generations 3 were 3-weeks-old AA broilers orally infected with sporulated EmiChIL-2 oocysts orally.*

*^b^Eighteen hours after inoculation, the chickens with transfected sporozoites were began a standard diet, supplemented with 150 ppm pyrimethamine.*

### Genome Walking

To validate the integration of the DNA fragment into the genome of transgenic *E. mitis*, genomic DNA was isolated from sporulated transgenic oocysts by phenol/chloroform extraction and ethanol precipitation (Yan et al., 2009) and the flanking sequences to the 5′ end of the integrated DNA were detected by using a Genome Walking Kit (Takara, Dalian, China). Specific reverse primers, SP1, SP2, and SP3 (**Table 1**) were designed according to histone 4 promoter sequence as per kit instructions. After the PCR amplification, the third-round PCR products were selected and cloned into the pEASY-T1 vector (TransGen Biotech, Beijing, China). The resulting putative sequences, confirmed by DNA sequencing, were then analyzed.

### Western Blot Analysis

To validate the expression of ChIL-2 in the transgenic *E. mitis*, Western blot analysis was performed as previously described (Liu et al., 2013). Briefly, soluble proteins extracted from EmiChIL-2 and the wild-type *E. mitis* in addition to the rChIL-2 as positive control, were resolved by SDS-PAGE and the immunoblot analysis following standard protocols. The primary antibody in this assay was the mouse anti-ChIL-2 polyclonal antibody, while the HRP-conjugated goat anti-mouse IgG (Proteintech, USA) was used as the secondary antibody.

### Indirect Immunofluorescence Assay (IFA)

In order to analyze the distribution of ChIL-2 in transgenic *E. mitis* sporozoites, IFA was preformed as previously described (Huang et al., 2011). Briefly, sporozoites of EmiChIL-2 and the wild-type *E. mitis* were, respectively, applied onto poly-L-lysine-coated slides. The primary and secondary antibodies in this assay were the mouse anti-ChIL-2 polyclonal antibody and the Cy3-conjugated goat anti-mouse IgG (Proteintech, USA), respectively.

### Measurement the Reproduction of EmiChIL-2

Twelve 1-week-old AA broilers were divided randomly into three groups to evaluate the reproduction of the transgenic parasites. Each bird in the different groups was orally inoculated with 1000 *E. mitis* oocysts suspended in 200 μl PBS, 1000 EmiChIL-2 oocysts suspended in 200 μl PBS, and 200 μl PBS (as control), respectively. Fecal samples were collected every 24 h from 3 to 11 dpi. The count of oocysts shed in the feces was determined by using McMaster egg counting chamber (Jeffers, 1975; Haug et al., 2006).

### Enzyme-Linked Immunospot Assay (ELISPOT)

To assess the cell-mediated immune response elicited by the EmiChIL-2 infection in chickens, twelve 3-weeks-old SPF chickens were divided randomly into three groups, and each of them was orally inoculated with 10^4^ of the wild-type *E. mitis* oocysts suspended in 200 μl PBS, 10^4^ EmiChIL-2 oocysts suspended in 200 μl PBS, and 200 μl PBS (as control) in the different groups, respectively. Gamma interferon (IFN-γ) secretion lymphocyte in the peripheral blood mononuclear cells (PBMCs) of chickens were analyzed 4 weeks after the immunization by ELISPOT, as previously described (Yin et al., 2013). Briefly, 10^6^ PBMCs from the PBS, the wild-type *E. mitis* and the EmiChIL-2 oocysts-immunized birds were stimulated with 10 μl PBS, 10 μl *E. mitis* oocysts antigen (10 μg/ml), and 10 μl PMA plus ionomycin (10 ng/ml PMA plus ionomycin 5 μg/ml), respectively. The spots in which IFN-γ secretion lymphocyte was present were detected after 24-h stimulation, as described by Yin et al. (2013).

### ELISA

To evaluate the humoral immune response stimulated by EmiChIL-2, twelve 1-week-old AA broilers were divided randomly into three groups, and each bird in the different groups was subjected to primary immunization with 1000 *E. mitis* oocysts suspended in 200 μl PBS, 1000 EmiChIL-2 oocysts suspended in 200 μl PBS and 200 μl PBS (as control) via oral route, respectively. Two weeks later, the immunized birds were boosted with 10,000 *E. mitis* oocysts suspended in 200 μl PBS, 10,000 EmiChIL-2 oocysts suspended in 200 μl PBS and 200 μl PBS (as control). The IgY (IgG) antibody titer in the serum was analyzed by ELISA, as reported previously (Huang et al., 2011). In brief, 5 μg/ml *E. mitis* oocysts antigen were coated onto the individual wells of the plates, followed by a reaction with serum (1:100) collected 2 weeks after the primary and boost immunization. The secondary antibody used in this experiment was the HRP-conjugated goat anti-chicken IgY Fc fragment (Bethyl Laboratories, Inc.).

### Wild Type *E. mitis* Challenge

To test the protective immunity stimulated by EmiChIL-2 vaccination against wild type *E. mitis* infection, 30 3-days-old AA broilers were divided into three groups, non-immunized (Ctrl), the wild-type *E. mitis*-immunized (500 oocysts/bird, WT) and EmiChIL-2-immunized (500 oocysts/bird, EmiChIL-2) group, respectively. Chickens were housed in the same condition. New litter of chopped straw was spread over cages’ bottom 5 cm, and chickens were fed a coccidian-free diet and water *ad libitum*. All the chickens were removed to new cages and challenged with the wild type *E. mitis* (10^4^ oocysts/bird) at 21 dpi. Fecal and litter samples were detected every 48 h post immunization, and oocysts shedding was measured by McMaster chamber (Jeffers, 1975; Haug et al., 2006).

### Statistical Analysis

Data were analyzed using the SPSS 12.0 (SPSS Institute Inc.). Differences in experimental treatments were tested using Duncan’s Multiple Range Test following ANOVA with significance reported at *P* ≤ 0.05.

## Results

### Construction of Transgenic *E. mitis* Expressing ChIL-2 (EmiChIL-2)

To construct a transgenic *E. mitis* secreting ChIL-2, we adapted the double expression-cassette plasmid, pHDEAAssChIL-2A (**Figure 1A**), in which, ChIL-2 was driven by an actin promoter. After nucleofection, EYFP was observed in transgenic sporozoites (**Figure 1Ba**) 24 h *in vitro.* Then, after six continuous passages (**Table 2**) under the action of pyrimethamine, we obtained a positive population with more than 90% expressing EYFP (**Figure 1Bb**).

To confirm that we successfully produced a stable transfected EmiChIL-2, we verified by PCR (**Figure 1C**) that the plasmid was introduced in the parasite genome and by genome walking that the integration site was Emh_scaff1365 (**Figures 1D,E**). The results from the Western blot assay (**Figure 1F**) and the IFA (**Figure 1G**) further evidenced that ChIL-2 was expressed and secreted by EmiChIL-2, respectively. Taken these data together, to further evaluate the potential as a novel coccidiosis vaccine component, we obtained a transgenic *E. mitis* population secreting ChIL-2.

### Reproduction of EmiChIL-2 vs. the Wild-Type *E. mitis*

**FIGURE 2:**
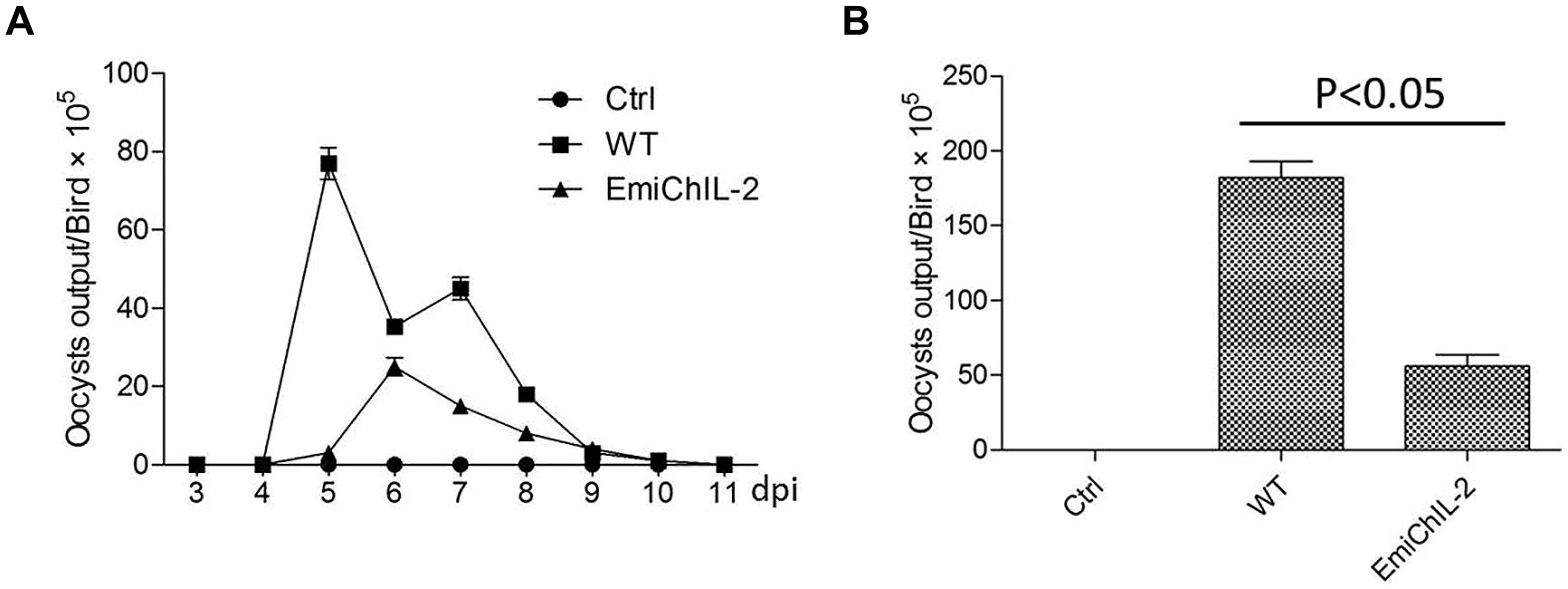
**Oocysts shedding of EmiChIL-2. (A)** Oocyst shedding was measured every 24 h from 3 to 11 dpi, and the mean value was estimated from four individuals. The wild-type *E. mitis* infection was used as a control. **(B)** Mean total oocyst output per chicken of EmiChIL-2 was three times lower than that of the wild-type *E. mitis* (*p* < 0.05) 3 to 11 days after the infection.

Reproduction, which reflects the parasite biological features and host resistance, was evaluated by the oocyst shedding dynamics (**Figure 2A**) and the total oocyst output (**Figure 2B**). The reproduction of EmiChIL-2 was substantially lower than that of the wild-type *E. mitis* as the total oocyst output per bird of EmiChIL-2 was reduced threefold relative to the wild-type *E. mitis* (**Figure 2B**). Meanwhile, the peak of oocyst shedding of EmiChIL-2 delayed about 24 h compared with that of the wild-type *E. mitis* (**Figure 2A**).

### EmiChIL-2 Stimulated a Higher Cellular Immune Response Compared with the Wild-Type *E. mitis*

**FIGURE 3:**
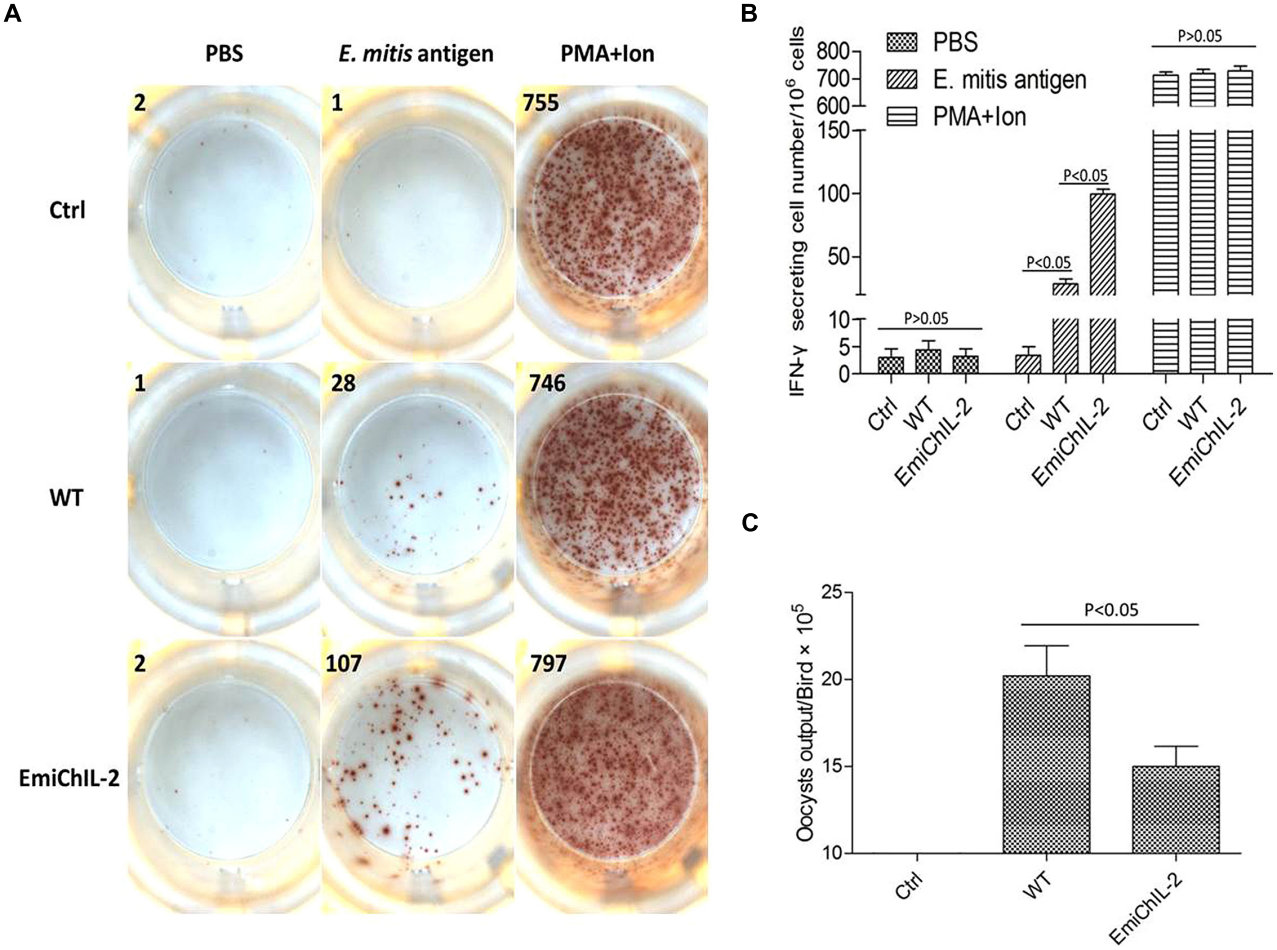
**Gamma interferon (IFN-γ) secretion lymphocytes in peripheral blood mononuclear cells (PBMCs) after immunization. (A)** 10^6^ PBMCs from PBS (Ctrl, upper), wild-type *E. mitis* (middle) and EmiChIL-2 (bottom) immunized birds (10^4^ oocysts/bird) were stimulated for 24 h with PBS (negative control, left), *E. mitis* oocysts antigen (middle) and PMA plus ionomycin (positive control, right). The number of IFN-γ secretion lymphocytes (spots) was determined as described in the section of materials and methods. **(B)** The mean amount of IFN-γ secretion lymphocytes in PBMCs in EmiChIL-2 immunized birds was significantly higher (*p* < 0.05) than that of the birds immunized with the wild-type *E. mitis* (*n* = 4). **(C)** Mean total oocyst output per chicken from 4 to 8 days after the immunization with EmiChIL-2 was much lower (*p* < 0.05) than after that, done with the wild-type *E. mitis*.

*Eimeria* sp. infection in chickens elicits both a humoral and cellular immune response, whereas the CMI plays a major role in the host protection against coccidiosis (Chapman et al., 2013). IFN-γ is an important component of the host protective CMI (Lillehoj and Choi, 1998). In this study, the IFN-γ secretion lymphocytes in PBMCs were analyzed post immunization by ELISPOT. As assumed in our hypothesis, the amount of *E. mitis*-specific IFN-γ secretion lymphocytes was significantly higher in PBMCs of EmiChIL-2 than those of the wild-type *E. mitis*-immunized birds (**Figures 3A,B**). The oocyst output of EmiChIL-2 was substantially lower (*p* < 0.05) than the obtained by the birds, immunized with the wild-type *E. mitis* (**Figure 3C**). Interestingly, the oocyst output from immunized birds with 10,000 oocysts of both EmiChIL-2 and the wild-type (**Figure 3C**) was much lower than that from 1000 oocysts-immunized birds (**Figure 2B**) as a result of ‘crowding effect’ (Williams, 2001). The above findings indicated that EmiChIL-2 stimulated a higher cellular immune response compared with the wild-type *E. mitis.*

### EmiChIL-2 Stimulated a Similar Humoral Immune Response Compared with the Wild-type *E. mitis*

**FIGURE 4:**
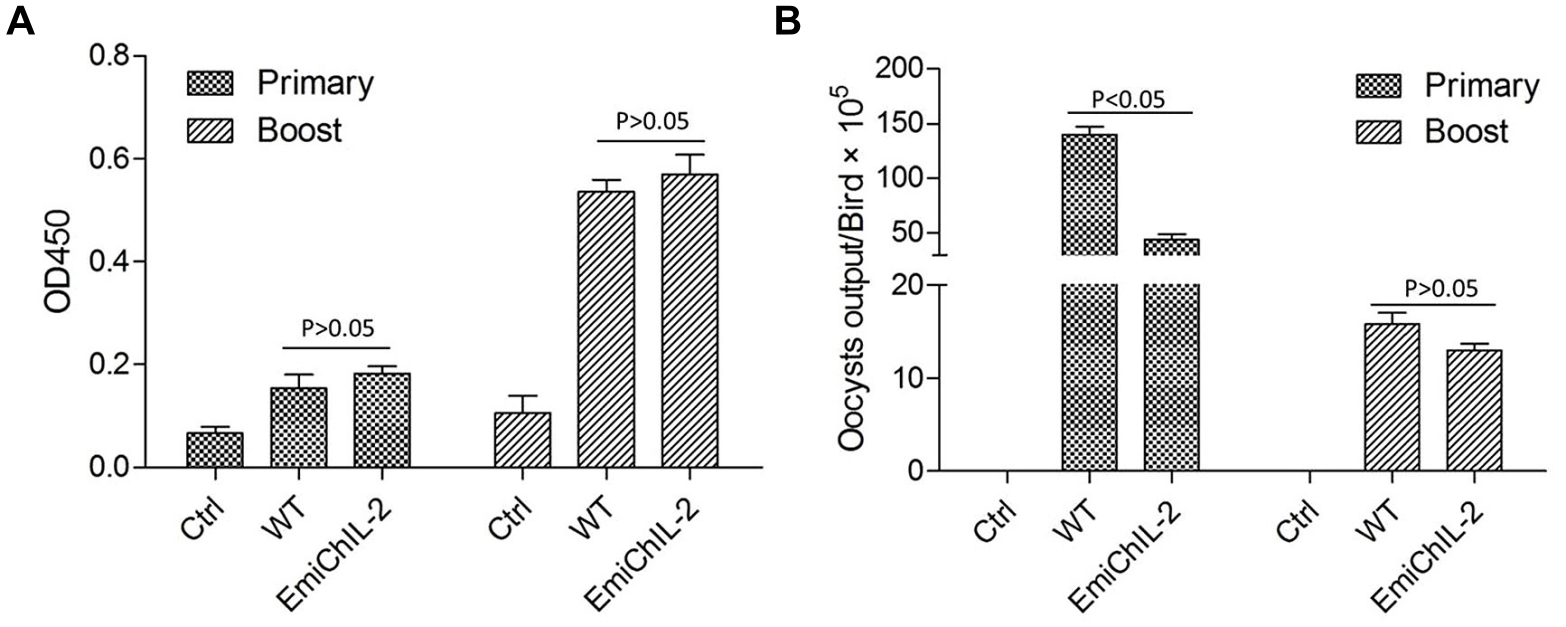
**IgY (IgG) antibody titer in the serum and oocyst output post primary and boost immunization. (A)** The OD_450_ value was similar between the birds immunized with EmiChIL-2 and those, immunized with the wild-type *E. mitis* 2 weeks after both the primary and the boost immunization (*p* > 0.05). **(B)** Mean total oocyst output per chicken of EmiChIL-2 was significantly lower than that of the wild-type *E. mitis* after the primary immunization (*p* < 0.05) 4–8 days, whereas, there was no significant difference between the EmiChIL-2 and the wild-type *E. mitis*-immunized birds after the boost immunization (*p* > 0.05) 4 to 8 days.

Interleukin 2 is a canonical T cell growth factor and plays a role in the expansion and differentiation of CD4+ T cells that help B lymphocytes secreting immunoglobulins (Zhang et al., 2014). Here, by the application of ELISA, we evaluated the humoral immune response, stimulated by EmiChIL-2 after the primary and boost immunization. However, there was no significant difference (*P* > 0.05) in the IgY titers in the serum after both the primary and the boost immunization of the birds, immunized with EmiChIL-2 and the wild-type *E. mitis* (**Figure 4A**). The reduced oocyst output of birds, immunized with EmiChIL-2 in comparison with that of the ones with immunization by the wild-type parasites (**Figure 4B**) may be due to CMI rather than on humoral immunity.

### EmiChIL-2 Enhances Protection of Chickens against Wild Type *E. mitis* Challenge

**FIGURE 5:**
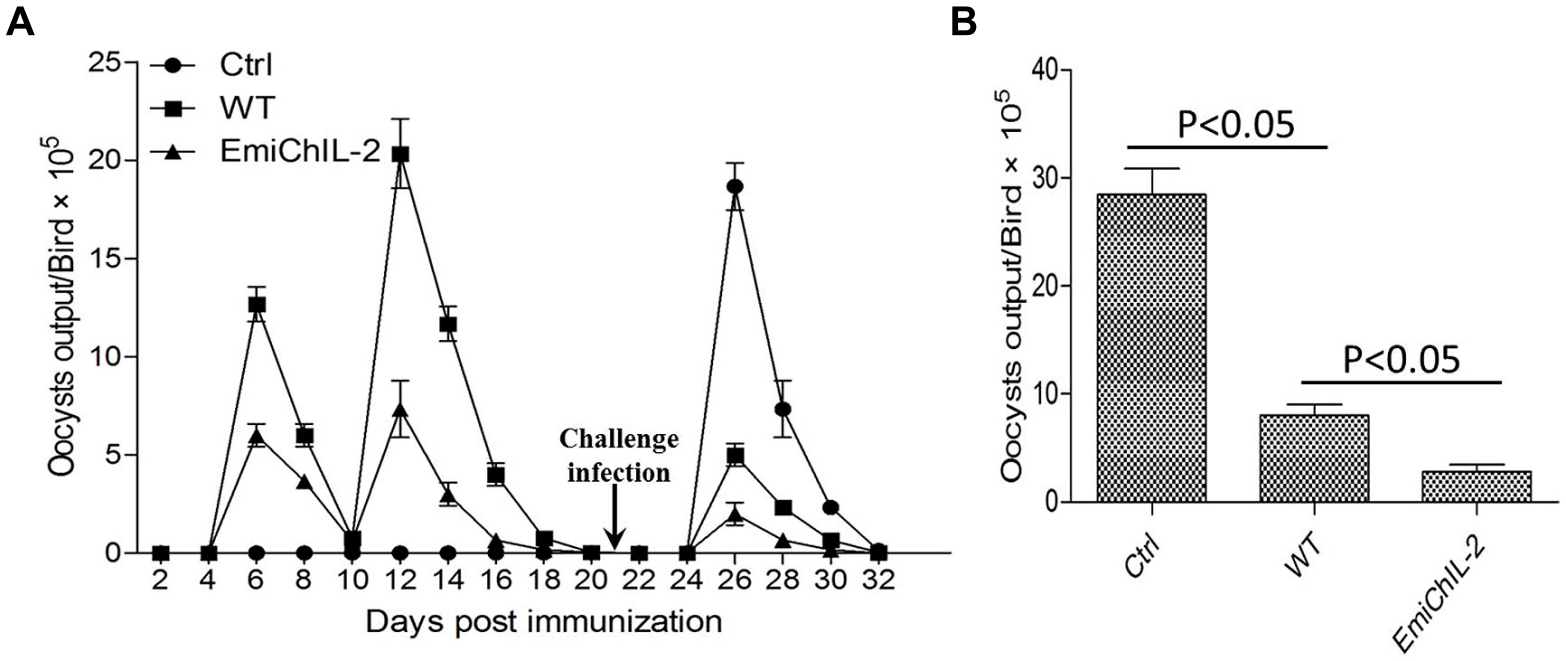
**Oocyst output following challenge infection in birds vaccinated with or without EmiChIL-2 or its wild type. (A)** Oocysts in fecal and litter samples (*n* = 10) every 2 days post vaccination with transgenic or wild type *E. mitis* and challenge with wild-type *E. mitis* (10^4^ oocysts/bird). **(B)** Mean total oocyst output per chicken between 4 and 8 days post challenge.

Immunization of chickens with anticoccidial vaccines elicits immunity against *Eimeria* sp. infection, and the immunity is automatically boosted when vaccinated chickens ingest offspring oocysts excreted into litter (Jeffers, 1975; Shirley et al., 2005; Chapman et al., 2013). We examined whether EmiChIL-2 enhanced protection of chickens against wild type *E. mitis* challenge with oocyst output reduction as a parameter of protection. EmiChIL-2 immunized chickens excreted fewer oocysts as compared with wild type-immunized chickens post challenge with wild type *E. mitis* (**Figures 5A,B**), indicating ChIL-2 enhanced the immunogenicity of the transgenic parasite.

## Discussion

Here, we demonstrated that transgenic *E. mitis* expressing secreted ChIL-2 induced a higher cellular immune response in chickens than did the wild-type *E. mitis*. Moreover, the reproduction of the transgenic parasite was reduced significantly. We speculated that the enhanced cellular immune response stimulated by EmiChIL-2, which rapidly eliminated the intracellular pathogens, contributed mainly to the reduced reproduction of EmiChIL-2. The expression of exogenous proteins and their toxicity to the parasite might have also influenced in part the biology features of the parasite, resulting in the lowered reproduction of EmiChIL-2 (Yan et al., 2009; Huang et al., 2011). In addition, the expression of EYFP in transgenic *E. tenella* and *E. mitis* lines elicited EYFP-specific immune response but did not alter the *Eimeria*-specific immune response (Huang et al., 2011; our unpublished data). So, the effect of enhanced *Eimeria*-specific cellular immune response in this study was interpreted here as being due to ChIL-2 secreted by EmiChIL-2.

Interleukin 2 continuously expressed by *L. lactis* or by a co-injection with CpG in a melanoma infection model significantly enhanced both humoral and cellular immune responses in mice with only one treatment (Addison et al., 1998; Zhang et al., 2014). In an *in vitro* cell model, continuous IL-2 secretion is required for enhancing activation of CD8+ T cell (Steenblock et al., 2011). These are in consistence with our EmiChIL-2 model, where ChIL-2 was driven by an actin promoter, a house-keeping gene promoter, guaranting that ChIL-2 was expressed and secreted into the parasite infection immune microenvironment continuously during the whole life cycle (Zou et al., 2009; Yin et al., 2011).

The finding that IL-2 expressing *E. mitis* obtained higher immunogenicity and protected chickens from wild type *E. mitis* infection suggested that the transgenic *Eimeria* sp. expressing cytokines, such as ChIL-2, could be utilized as an highly effective anticoccidial vaccine strains, encouraging our further transgenesis of IL-2 in some other *Eimeria* sp., such as *E. tenella* and *E. necatrix*, which are highly pathogenic but have intermediate immunogenicity. Enhanced immunogenicity helps vaccinated chickens develop solid immunity quickly, preventing vaccination side effect through preventing re-infections. We conclude that transgenic attenuated anticoccidial vaccine strains are promising for their safe application in broilers.

## Conflict of Interest Statement

The authors declare that the research was conducted in the absence of any commercial or financial relationships that could be construed as a potential conflict of interest.

## References

[B1] Addison C. L. Bramson J. L. Hitt M. M. Muller W. J. Gauldie J. Graham F. L. (1998). Intratumoral coinjection of adenoviral vectors expressing IL-2 and IL-12 results in enhanced frequency of regression of injected and untreated distal tumors. *Gene Ther.* 5 1400–1409. 10.1038/sj.gt.3300731 9930346

[B2] Blachere N. E. Morris H. K. Braun D. Saklani H. Di Santo J. P. Darnell R. B. (2006). IL-2 is required for the activation of memory CD8+ T cells via antigen rross-presentation. *J. Immunol.* 176 7288–7300. 10.4049/jimmunol.176.12.7288 16751372

[B3] Chapman H. D. (2000). Practical use of vaccines for the control of coccidiosis in the chicken. *Worlds Poult. Sci. J.* 56 7–20. 10.1079/WPS20000002

[B4] Chapman H. D. Barta J. R. Blake D. Gruber A. Jenkins M. Smith N. C. (2013). A selective review of advances in coccidiosis research. *Adv. Parasitol.* 83 93–171. 10.1016/B978-0-12-407705-8.00002-1 23876872

[B5] Clark J. D. Billington K. Bumstead J. M. Oakes R. D. Soon P. E. Sopp P. (2008). A toolbox facilitating stable transfection of *Eimeria* species. *Mol. Biochem. Parasitol.* 162 77–86. 10.1016/j.molbiopara.2008.07.006 18723051

[B6] Danforth H. D. (1998). Use of live oocyst vaccines in the control of avian coccidiosis: experimental studies and field trials. *Int. J. Parasitol.* 28 1099–1109. 10.1016/S0020-7519(98)00078-72 9724881

[B7] Grimm E. A. Mazumder A. Zhang H. Z. Rosenberg S. A. (1982). The lymphokine activated killer cell phenomenon. Lysis of natural killer resistant fresh solid tumor cells by interleukin-2 activated autologous human peripheral blood lymphocytes. *J. Exp. Med.* 155 1823–1841. 10.1084/jem.155.6.1823 6176669 PMC2186695

[B8] Haug A. Williams R. B. Larsen S. (2006). Counting coccidial oocysts in chicken faeces: a comparative study of a standard McMaster technique and a new rapid method. *Vet. Parasitol.* 136 233–242. 10.1016/j.vetpar.2005.11.024 16388903

[B9] Huang X. Zou J. Xu H. Ding Y. Yin G. Liu X. (2011). Transgenic *Eimeria tenella* expressing enhanced yellow fluorescent protein targeted to different cellular compartments stimulated dichotomic immune responses in chickens. *J. Immunol.* 187 3595–3602. 10.4049/jimmunol.1100043 21876035

[B10] Jeffers T. K. (1975). Attenuation of *Eimeria tenella* through selection for precociousness. *J. Parasitol.* 61 1083–1090. 10.2307/3279381 1195070

[B11] Lillehoj H. S. Choi K. D. (1998). Recombinant chicken interferon-gamma-mediated inhibition of *Eimeria tenella* development in vitro and reduction of oocyst production and body weight loss following *Eimeria acervulina* challenge infection. *Avian. Dis.* 42 307–314. 10.2307/1592481 9645322

[B12] Liu X. Zou J. Yin G. Su H. Huang X. Li J. (2013). Development of transgenic lines of *Eimeria tenella* expressing M2e-enhanced yellow fluorescent protein (M2e-EYFP). *Vet. Parasitol.* 193 1–7. 10.1016/j.vetpar.2012.12.019 23298569

[B13] Long P. L. Millard B. J. Joyner L. P. Norton C. C. (1976). A guide to laboratory techniques used in the study and diagnosis of avian coccidiosis. *Folia Vet. Lat.* 6 201–217. 1010500

[B14] Pardoll D. M. (2002). Vaccines: spinning molecular immunology into successful immunotherapy. *Nat. Rev. Immunol.* 2 227–238. 10.1038/nri774 12001994

[B15] Rochman Y. Spolski R. Leonard W. J. (2009). New insights into the regulation of T cells by γc family cytokines. *Nat. Rev. Immunol.* 9 480–490. 10.1038/nri2580 19543225 PMC2814538

[B16] Shi T. Yan W. Ren H. Liu X. Suo X. (2009). Dynamic development of parasitophorous vacuole of *Eimeria tenella* transfected with the yellow fluorescent protein gene fused to different signal sequences from apicomplexan parasites. *Parasitol. Res.* 104 315–320. 10.1007/s00436-008-1194-y 18815811

[B17] Shirley M. W. Smith A. L. Tomley F. M. (2005). The biology of avian *Eimeria* with an emphasis on their control by vaccination. *Adv. Parasitol.* 60 285–330. 10.1016/S0065-308X(05)60005-X 16230106

[B18] Steenblock E. R. Fadel T. Labowsky M. Pober J. S. Fahmy T. M. (2011). An artificial antigen-presenting cell with paracrine delivery of IL-2 impacts the magnitude and direction of the T cell response. *J. Biol. Chem.* 286 34883–34892. 10.1074/jbc.M111.276329 21849500 PMC3186438

[B19] Suo X. Zhang J. X. Li Z. G. Yang C. T. Min Q. R. Xu L. T. (2006). The efficacy and economic benefits of Supercox^®^, a live anticoccidial vaccine in a commercial trial in broiler chickens in China. *Vet. Parasitol.* 142 63–70. 10.1016/j.vetpar.2006.06.020 16876953

[B20] Trinchieri G. (1989). Biology of natural killer cells. *Adv. Immunol.* 47 187–376. 10.1016/S0065-2776(08)60664-1 2683611 PMC7131425

[B21] Williams R. B. (1998). Epidemiological aspects of the use of live anticoccidial vaccines for chickens. *Int. J. Parasitol.* 28 1089–1098. 10.1016/S0020-7519(98)00066-6 9724880

[B22] Williams R. B. (2001). Quantification of the crowding effect during infections with the seven *Eimeria* species of the domesticated fowl: its importance for experimental designs and the production of oocyst stocks. *Int. J. Parasitol.* 31 1056–1069. 10.1016/S0020-7519(01)00235-1 11429169

[B23] Yan W. Liu X. Shi T. Hao L. Tomley F. M. Suo X. (2009). Stable transfection of *Eimeria tenella*: constitutive expression of the YFP-YFP molecule throughout the life cycle. *Int. J. Parasitol.* 39 109–117. 10.1016/j.ijpara.2008.06.013 18718473

[B24] Yin G. Liu X. Zou J. Huang X. Suo X. (2011). Co-expression of reporter genes in the widespread pathogen *Eimeria tenella* using a double-cassette expression vector strategy. *Int. J. Parasitol.* 41 813–816. 10.1016/j.ijpara.2011.04.001 21550346

[B25] Yin G. Qin M. Liu X. Suo J. Suo X. (2013). Interferon-gamma enzyme-linked immunosorbent spot assay as a tool to study T cell responses to *Eimeria tenella* infection in chickens. *Poult. Sci.* 92 1758–1763. 10.3382/ps.2012-8 23776262

[B26] Zhang H. Qiu Y. Zhao Y. Liu X. Liu M. Yu A. (2014). Immunogenicity of oral vaccination with *Lactococcus lactis* derived vaccine candidate antigen (UreB) of *Helicobacter pylori* fused with the human interleukin 2 as adjuvant. *Mol. Cell Probe* 28 25–30. 10.1016/j.mcp.2013.08.003 24036137

[B27] Zou J. Liu X. Shi T. Huang X. Wang H. Hao L. (2009). Transfection of *Eimeria* and *Toxoplasma* using heterologous regulatory sequences. *Int. J. Parasitol.* 39 1189–1193. 10.1016/j.ijpara.2009.03.006 19379753

